# Au Doping PtNi Nanodendrites for Enhanced Electrocatalytic Methanol Oxidation Reaction

**DOI:** 10.3390/nano13212855

**Published:** 2023-10-28

**Authors:** Shan Wang, Lifeng Ma, Dan Song, Shengchun Yang

**Affiliations:** 1 Key Laboratory for Molecular Genetic Mechanisms and Intervention Research on High Altitude Disease of Tibet Autonomous Region, School of Medicine, Xizang Minzu University, No. 6 East Wenhui Road, Xianyang 712082, China; lfma@xzmu.edu.cn (L.M.); dsong@xzmu.edu.cn (D.S.); 2 Ministry of Education Key Laboratory for Non-Equilibrium Synthesis and Modulation of Condensed Matter, Key Laboratory of Shaanxi for Advanced Materials and Mesoscopic Physics, State Key Laboratory for Mechanical Behavior of Materials, School of Physics, Xi’an Jiaotong University, No. 28 West Xianning Road, Xi’an 710049, China; 3 National Innovation Platform (Center) for Industry-Education Integration of Energy Storage Technology, Xi’an Jiaotong University, No. 28 West Xianning Road, Xi’an 710049, China; 4 Shaanxi Collaborative Innovation Center for Hydrogen Fuel Cell Performance Improvement, Xi’an Jiaotong University, No. 28 West Xianning Road, Xi’an 710049, China

**Keywords:** Au dopant, nanodendrite, AuPtNi shell, methanol oxidation reaction

## Abstract

To boost the electrocatalytic methanol oxidation reaction (MOR) of Platinum (Pt), making binary PtM (M = transition metals, for example, Fe, Cu, and Ni) with specific morphology is known as a promising method. Although great progress has been made in the synthesis of shaped PtM catalysts toward MOR, enhancing the catalytic performance of the PtM to enable it to be commercialized is still a hotspot. In this work, the Au-doped PtNi dendritic nanoparticles (Au-PtNi DNPs) were obtained by doping a small amount of gold (Au) into initially prepared PtNi DNPs, greatly improving their MOR catalytic activity and durability. The energy-dispersive X-ray spectroscopy mapping (EDXS) indicates that the surface of DNPs is mainly composed of Au dopant and PtNi, while the core is mainly Pt, indicating the formation of Au-doped PtNi/Pt core-shell-like DNP structures. The electrocatalytic performance of the prepared Au-PtNi DNPs with different compositions for the MOR was evaluated using cyclic voltammetry, chronoamperometry, and CO-stripping tests. The experimental findings indicate that the Au-PtNi DNPs showed better MOR performance in comparison with PtNi DNPs and commercial Pt catalysts. Among all the catalysts, 6% Au-PtNi DNPs showed 4.3 times improved mass catalytic activity for the MOR in comparison with commercial Pt catalysts. In addition, all the prepared Au-PtNi DNPs display a remarkable CO tolerance compared to that of PtNi DNPs and commercial Pt catalysts. The dendritic structure of Au-PtNi DNPs can effectively enhance catalytic performance, combined with the electronic effect of Au, Pt, and Ni.

## 1. Introduction

Direct methanol fuel cells (DMFCs) are well on the way to becoming promising energy conversion devices due to their environmental friendliness, portability, safety to operate, high energy density, and transfer rate. DMFCs are still considered an ideal concept for future electronic product development because they deal with liquid fuel [1,2,3]. The development of DMFCs largely relies on the methanol oxidation reaction (MOR), while catalysts are considered to be critical factors in the process. It is well known that Platinum (Pt) is regarded as the most effective element in electrocatalysts for MOR; however, the high cost induced by limited reserves, sluggish MOR kinetics, and easy poisoning of Pt greatly impede the commercial development of DMFCs to becoming more widespread [4]. Therefore, the improvement of a per-Pt-atom catalytic activity should be necessary to solve above mentioned issues. Currently, alloying Pt with other transition metals (TMs, such as Ni, Co, Fe, Cu, and Mo) to produce PtM bimetal catalysts is considered an effective method to enhance electrocatalytic performance via adjusting the electronic structure (or d-band center position) of Pt [5,6,7]. Furthermore, the surface structure of a PtM electrocatalyst, which largely rests with the morphology and architecture, is another critical factor that influences the electrocatalytic performance [8,9]. Recently, methods combining the benefits of “electronic effects” and “surface effects” to achieve an improved performance of PtM electrocatalysts have been recognized as the optimal strategy to designed electrocatalysts. PtM binary electrocatalysts with various morphologies—such as polyhedron [10], nanowire [11,12], nanoframe [13], porous [14], and core-shell structure [15]—are considered effective electrocatalysts attributed to their unique surface structures. Among the various explored PtM binary electrocatalysts, the PtNi catalyst is the most promising in enhancing electrocatalytic behavior toward MOR [16,17]. 

However, in practice, the element segregation and the longtime electrochemical-corrosion-induced PtNi catalyst lose their high intrinsic electrocatalytic activity; moreover, the aggregation of harmful intermediate products on the catalyst surface during the electrocatalytic reaction leads to poisoning and inactivation of the PtNi catalyst. The degradation of the surface composition and structure of PtNi nanoparticles (NP) is the main reason for the decrease in the activity of PtNi catalysts. Recently, some research suggested that introducing a small quantity of another element to the PtM can increase the structural and electronic stability of the PtNi nanoparticles, thus enhancing the electrocatalytic activity and durability [6,18,19]. The element gold (Au) as a catalyst component is inert in electrolytes, which can stabilize the morphology of the Pt-based nanoparticles, thus enhancing their electrochemical stability and durability [20,21,22]. Furthermore, Au can modify the electronic structure of the Pt to improve its electrocatalytic performance by weakening the CO or CO-like intermediary species bound to the surface of Pt in the MOR reaction [23,24]. Recently, PtAu [24], AuPtNi [23], NiAuPt-NGs [25], PtPdAu/graphene [26], AuPtRh [27], Au-PtCu [14], Au-PtCo/C [28], Pt@Au_x_Cu_100−x_ [29], and Au@PtCu [30] catalysts with much-enhanced performances for MOR, ethanol oxidation reaction (EOR), formic acid electrooxidation, and oxygen reduction reaction (ORR) have been successfully prepared. For example, Huang et al. confirmed that the tensile stress around the Pt-Au interfaces and the electrons transferring from the Pt to Au can assist in the oxidation of CO or CO-like intermediate species, thus improving the MOR performance of Pt-Au heteronanowires [24]. Zhang et al. found that Au played the role of electronic modifier, increasing the CO tolerance of Pt by weakening the CO binding on Pt in AuPtNi nanostructures, thus enhancing their MOR and EOR activities [23]. Kuttiyiel et al. have successfully synthesized Au-doped L_10_ PtCo ordered intermetallic nanoparticles. They concluded that the dopant Au can not only prevent the oxidation of Pt and the dissolution of Co but can also decorate the Pt surface to decrease the binding energy of O and OH and, therefore, enhance the electrocatalytic behavior of PtCo for ORR [28]. Xie et al. confirmed that Au could upshift the Pt d-band center, and stabilize the surface of the Pt_37_Cu_56_Au_7_ porous film catalyst [14]. Although Au-alloyed metal catalysts for a few reactions have been widely studied, the impact of Au doping into PtNi catalysts with specific morphology for the MOR is still scarcely reported. It has been known that PtNi catalysts exhibit better electrocatalytic performance toward the MOR, which has stimulated our research on doping Au in shaped PtNi catalysts and investigating their electrocatalytic activity and durability toward the MOR. To further increase the reaction active sites of PtM catalysts and synchronously enhance their electrocatalytic performance, nanodendrites with a three-dimensional (3D) porous structure have attracted much attention. The dendritic structure can provide numerous low-coordinated atomic steps, edges, thorns, and abundant electron transfer pathways, presenting electrons and geometric structures that are conductive to the MOR reaction [31,32].

In this study, the Au-doped PtNi dendritic nanoparticles (Au-PtNi DNPs) with different contents of Au were synthesized using a simple two-step strategy. Firstly, PtNi dendritic nanoparticles (PtNi DNPs) with a PtNi shell and Pt core were used for the starting material, as described in our previous report [32]. Then, Au was doped in a PtNi shell via an in situ reduction of Au^3+^ using a one-pot method. Compared with PtNi DNPs and commercial Pt, Au-doped PtNi DNPs significantly improve their electrocatalytic performance for the MOR. The research indicates that the 6% Au-PtNi DNPs (6 at.% Au) can be a promising substitute for low-Pt MOR electrocatalysts.

## 2. Experimental Design

### 2.1. Reagents and Chemicals

Chloroauric acid [HAuCl_4_·4H_2_O, AR.] and platinum (II) acetylacetonate [Pt(acac)_2_, AR.] were bought from Kunming Institute of Precious Metals. Polyvinylpyrrolidone (PVP, MW of ~55,000), sulfuric acid [H_2_SO_4_, AR.], and Nickel (II) acetylacetonate [Ni(acac)_2_, AR.] were bought from Sinopharm Chemical Reagent Co., Ltd. (Shanghai, China). Ethylene glycol (EG, AR.), Phemethylol (AR.), Chlorhydric acid (HCl, AR.), Nitric acid (HNO_3_, AR.), and Ethanol (AR.) were obtained from Tianjin Tian Li Chemical Reagent Co., Ltd. (Tianjin, China). Methyl alcohol (CH_3_OH, 99.9%) was purchased from Aladdin Reagent Co., Ltd. (Shanghai, China). Deionized (DI) water (18.25 MΩ/cm) needed in this work was prepared using an ultrapure purification system. 

### 2.2. Preparation of Au-PtNi DNPs

PtNi DNPs were synthesized according to the procedure in our previous report [32]. In brief, 8 mg of Pt(acac)_2_, 1.75 mg of Ni(acac)_2_, 40 mg of PVP, 50 µL of 5 M HCl/H_2_O, 40 µL of 2 M HNO_3_/H_2_O solution, and 5 mL of phemethylol were added into a flask (25 mL) and stirred until well-mixed. Then, the above mixture was heated to 150 °C with magnetic stirring under the oil bath for 6 h and the black PtNi DNP slurry was obtained. Finally, 5 mL of PtNi DNP slurry was taken out and centrifugated utilizing water/ethanol two times and redispersed in 5 mL of EG as starting material for further use.

For prepared Au-PtNi DNPs, 5 mL of the prepared-starter PtNi DNPs was moved into 25 mL flask and heated to 150 °C with magnetic stirring under an oil bath. Then, 2 mL of 2 M HAuCl_4_/H_2_O was put into the above starter PtNi DNPs under magnetic stirring and further heated at 150 °C for 30 min. After cooling down the reaction slurry to room temperature, the prepared black slurry was centrifugated using water/ethanol for three times. Finally, the 6% Au-PtNi DNPs (6 at.% Au) were obtained and redispersed in water for later use. Using the same procedure, 2% Au-PtNi DNPs (2 at.% Au) and 11% Au-PtNi DNPs (11 at.% Au) were synthesized by altering the added HAuCl_4_/H_2_O solution to 1 mL and 4 mL, respectively.

### 2.3. Electrochemical Measurements

Typically, the obtained Au-PtNi DNPs were dispersed in 2 mL of distilled water and 0.5 mL of 2-propanol, followed by ultrasonic treatment for 15 min to prepare catalyst ink. Then, 5 μL of the catalyst ink was cast on a precleaned glassy carbon electrode (GCE), followed by drying at 30 °C. The diameter of glassy carbon in GCE was 5 mm (geometric area = 0.196 cm^2^). Electrochemical tests were proceeded on a Pine AFCBP1 Electrochemical Analyzer Instrument. A graphite rod counter electrode and a Ag/AgCl (in saturated KCl, aq) reference electrode were combined with a GCE working electrode to assemble a classical three-electrode cell.

Before measurement, ~25 cycles were performed in nitrogen (N_2_)-purged 0.5 M H_2_SO_4_ aqueous solution from −0.21 to 0.99 V (vs. Ag/AgCl) with 150 mV/s until the stabilized cyclic voltammetry (CV) plots were acquired. The CV plots were acquired under the same conditions by altering the sweep rate to 50 mV/s. The electrochemical active surface area (ECSA) of the Au-PtNi DNPs was calculated by integrating the hydrogen adsorption area from the CV curves.

The electrocatalytic performance of MOR was evaluated under nitrogen-saturated 0.5 M H_2_SO_4_ aqueous solution containing 0.5 M CH_3_OH, and the test potential employed was from −0.2 to 0.99 V (vs. Ag/AgCl) with a scan rate of 50 mV/s for the CV tests. The steady CV plots were recorded after sweeping about 20 cycles. CA plots were performed under a N_2_-purged 0.5 M H_2_SO_4_ aqueous solution containing 0.5 M CH_3_OH at 0.6 V (vs. Ag/AgCl) for 3000 s.

The CO saturation coverage was carried out by putting the prepared working electrode into a CO-purged 0.5 M H_2_SO_4_ electrolyte with the potential of −0.114 V (vs. Ag/AgCl) for 10 min. Next, the CO-covered working electrode was transferred to N_2_-saturated 0.5 M H_2_SO_4_ aqueous solution to test CO-stripping curves from −0.21 to 0.99 V (vs. Ag/AgCl) with 50 mV/s for two cycles. The CO_ads_ monolayer electrooxidation required 420 μC/cm^2^ of Pt. The ECSA values of catalysts were also calculated from the CO stripping voltammograms [33].

In the same manner, the electrocatalytic performance of commercial Pt black (Alfa Aesar, Haverhill, MA, USA) on MOR was evaluated for comparison.

### 2.4. Characterization

The sizes and morphologies of NPs were analyzed using field emission scanning electron microscopy (FESEM, JSM-7000F, JEOL, Akishima, Japan) and a transmission electron microscope (TEM, JEM-2100, JEOL, Japan). High-angle annular dark-field scanning transmission electron microscopy (HAADF–STEM) combining EDXS (HAADF–STEM-EDXS) mapping were operated on JEM-F200 (JEOL, Japan). The crystalline structure of the NPs was obtained using an X-ray diffractometer (XRD, Bruker, Mannheim, Germany, AXS, Cu Kα radiation, λ = 0.15418 nm). The elemental constitution of the samples was recorded on an inductively coupled plasma atomic emission spectrometer (ICP-AES, ICPE-9000, Shimadzu, Kyoto, Japan). 

## 3. Results and Discussion

**Figure 1:**
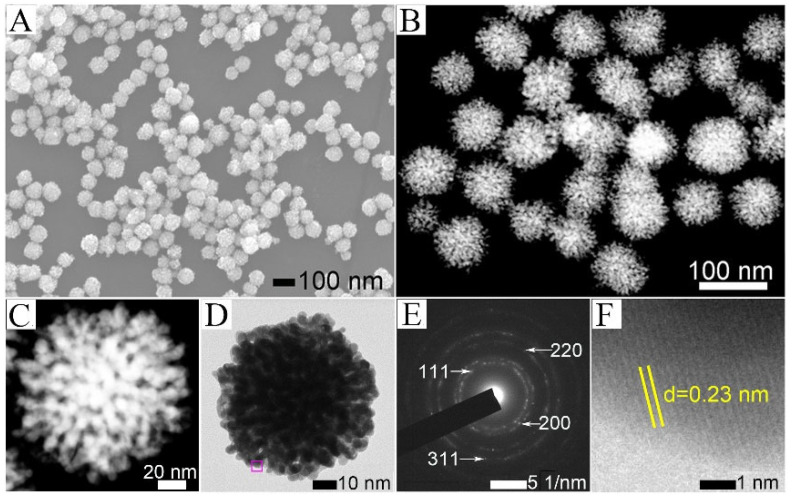
(**A**) SEM and (**B**) HAADF-STEM images of Au-PtNi DNPs. (**C**) HAADF-STEM and (**D**) TEM images of a single 6% Au-PtNi DNP. (**E**) SAED patterns of a single 6% Au-PtNi DNP in (**D**). (**F**) HR-TEM image of the circled region in (**D**).

The morphology and structure of 6% Au-PtNi DNPs prepared using the typical synthesis were studied using FESEM, HAADF-STEM, and TEM. The SEM image (Figure 1A) exhibits that the NPs have a uniform spherical-like shape. The average diameter of these Au-PtNi DNPs is ~73 nm (Figure S1A). Furthermore, the HAADF-STEM and TEM images of an individual DNP were also characterized to study the detailed structure. As shown in Figure 1B–D, the NPs show a 3D dendritic structure formed from numerous grains with a diameter of 3 nm. These DNPs can provide tremendous edges, thorns, and reaction-active sites, which are beneficial to the electrocatalytic reaction. The morphology and structure of Au-PtNi DNPs are consistent with the starting PtNi DNPs (Figure S2) prepared in our previous work [32], indicating that the morphology of the starting nanodendrite can be well maintained by doping Au on the surface of PtNi DNPs. Figure 1E reveals the selected area electron diffraction (SAED) pattern of an individual Au-PtNi DNP, which is consistent with (111), (200), (220), and (311) planes of the SAED pattern of Au-PtNi DNPs, corresponding to its face-centered cubic (fcc) structure, confirming good crystallization performance of Au-PtNi DNPs [34]. As shown in Figure 1F, the HR-TEM image exhibits the lattice fringes of 0.23 nm in the branch edge, which is higher than that of 0.22 nm in PtNi DNPs (Figure S3), which is assignable to the (111) planes of alloyed Au-PtNi. These results suggest that the Au can be successfully doped in PtNi DNPs, meanwhile, the dendritic morphology can be preserved well.

**Figure 2:**
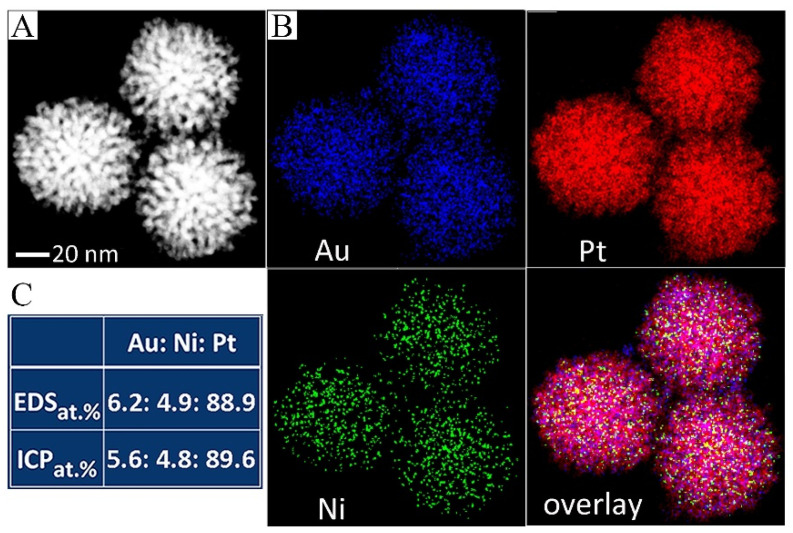
(**A**) HAADF–STEM image and (**B**) corresponding EDXS mapping of 6% Au-PtNi DNPs. (**C**) EDS analyses and ICP results of 6% Au-PtNi DNPs.

The element distribution of the prepared Au-PtNi DNPs was characterized by HAADF-STEM. The results display that the Au-PtNi DNPs possess a 3D porous structure composed of abundant branches (Figure 2A). The corresponding EDXS mapping of Au-PtNi DNPs is shown in Figure 2B. The EDXS mapping suggests that the dopants Au and Ni are mainly located on the surface of DNPs rather than inside, while Pt is uniformly distributed throughout the entire DNPs, indicating the formation of Au-PtNi NPs with a core-shell-like dendritic structure with AuPtNi as the shell and Pt as the core. The ICP-AES results present that Au:Ni:Pt has a 5.6:4.8:89.6 atomic ratio, which agrees with the EDS results (Au:Ni:Pt was 6.2:4.9:88.9, thus, it was defined as 6% Au-PtNi DNPs), as shown in Figure 2C and Figure S1B. The molar ratio of Au and Pt in the products was similarly consistent with the feeding ratio of Au^3+^ and Pt in PtNi DNPs, indicating that almost all of the Au^3+^ was successfully doped in PtNi DNPs through the in situ reduction. The atomic ratio of Ni and Pt in Au-PtNi DNPs (5:95) is similar to that in the initial PtNi DNPs (Au:Ni was 6:94), which further indicates that the Au was doped in PtNi DNPs by the in situ reduction rather than the replacement between Au^3+^ and Ni in the initial PtNi DNPs. As reported in our previous work, the starting PtNi DNPs show core-shell-like structures with PtNi dominating the surface of DNPs and Pt dominating the core of DNPs [32]. Then, the Au^3+^ was doped on the surface of PtNi DNPs through the in situ reduction, forming the Pt core @ AuPtNi shell core-shell-like dendritic structure.

**Figure 3:**
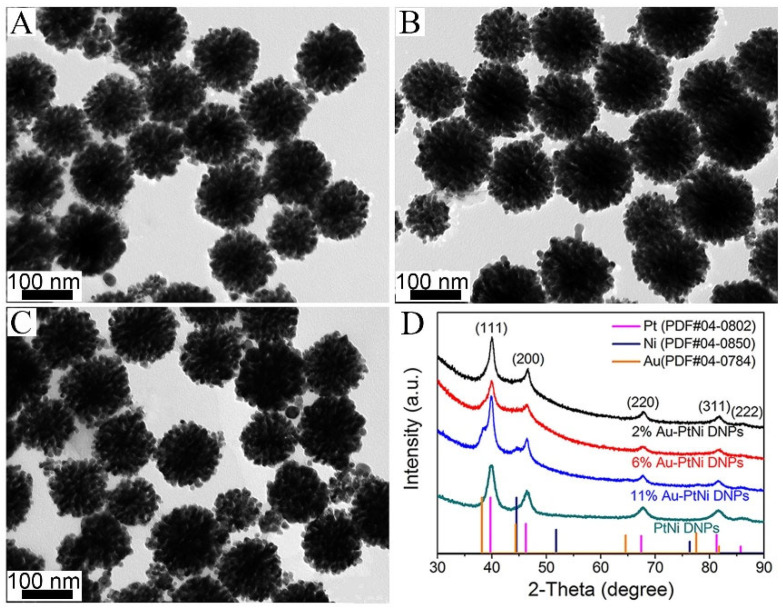
TEM images of (**A**) 2% Au-PtNi DNPs, (**B**) 6% Au-PtNi DNPs, and (**C**) 11% Au-PtNi DNPs. (**D**) XRD patterns of Au-PtNi and PtNi DNPs.

The content of the doping agent Au in the Au-PtNi DNPs can be adjusted by changing the volume of the HAuCl_4_ solution in the reaction. Figure 3A–C exhibit TEM images of Au-PtNi DNPs with various atomic ratios of Au, Pt, and Ni. When the feeding atomic ratio of HAuCl_4_ to Pt(acac)_2_ was increased from 2.5:100 (2% Au-PtNi DNPs) to 5:100 (6% Au-PtNi DNPs) and then to 10:100 (11% Au-PtNi DNPs), Au-PtNi NPs with an average diameter of ~73 nm and uniform dendritic morphology were obtained (Figures S1A, S3A and S4A). The composition of the Au-PtNi NPs was tested by ICP and EDS. The ICP and EDS results (Figures S1B, S3B and S4B) exhibit that the 2% Au-PtNi DNPs, 6% Au-PtNi DNPs, and 11% Au-PtNi DNPs can be prepared through changing the feeding atomic ratio of HAuCl_4_ and Pt(acac)_2_ from 2.5:100 to 5:100 and then to 10:100 while maintaining the same amount of Ni(acac)_2_. These characterizations show that the different content of Au can be doped in PtNi DNPs without changing the initial dendritic structure through the subsequent in situ reduction. Compared with initial PtNi DNPs, the proportion of doped Au in the Au-PtNi particles is small (up to 10 at.%), which is not enough to change the morphology of the starting PtNi DNPs. The dopant Au content in Au-PtNi DNPs can be adjusted by altering the addition of HAuCl_4_. The atomic ratios of Au and Pt in 2% Au-PtNi DNPs, 6% Au-PtNi DNPs, and 11% Au-PtNi DNPs were basically in agreement with the feeding ratio of Au^3+^ and Pt in PtNi DNPs, which further indicates that almost all the Au^3+^ in the reactants can be doped in PtNi DNPs through this in situ reduction method. The results further confirm that different amounts of Au can be doped into PtNi DNPs and preserve the unique three-dimensional dendritic structure, which remains unchanged by this simple two-step reduction strategy. The structure of Au-PtNi DNPs with various compositions and the initial material PtNi DNPs were analyzed using XRD (Figure 3D). The diffraction angles from AuPtNi DNPs and PtNi DNPs can be indexed to the (111), (200), (220), (311), and (222) planes of the face-centered cubic (fcc) structure of PtNi alloy and Au. A single set of diffraction peaks from 2% Au-PtNi DNPs and 6% Au-PtNi DNPs are situated within the region between pure Au (PDF#04-0784), Pt (PDF#04-0802), and Ni (PDF#04-0805) elements, indicating the formation of an alloy structure for 2% Au-PtNi DNPs and 6% Au-PtNi DNPs. When doped with a small amount of Au in the PtNi DNPs, such as 2% Au-PtNi DNPs or 6% Au-PtNi DNPs, the peak positions gradually shift toward lower angles, which demonstrates an increase in lattice spaces. This is in agreement with the HRTEM analysis in Figure 1F. At the same time, the as-prepared 11% Au-PtNi DNPs show two tiny peaks at 46.34° and 67.61°, corresponding to the {111} and {200} planes with the fcc structure of pure Au, indicating the formation of a Au cluster in AuPtNi DNPs. When PtNi DNPs react with a small amount of HAuCl_4_ solution, Au^3+^ can form a AuPtNi alloy on the surface of PtNi DNPs, which was also confirmed via the characterization results of the HAADF-STEM-EDX mapping. When there is more HAuCl_4_ solution in the system, the reaction continues, and the generated Au atoms are deposited on the AuPtNi, forming Au clusters on the surface of PtNi DNPs.

**Figure 4:**
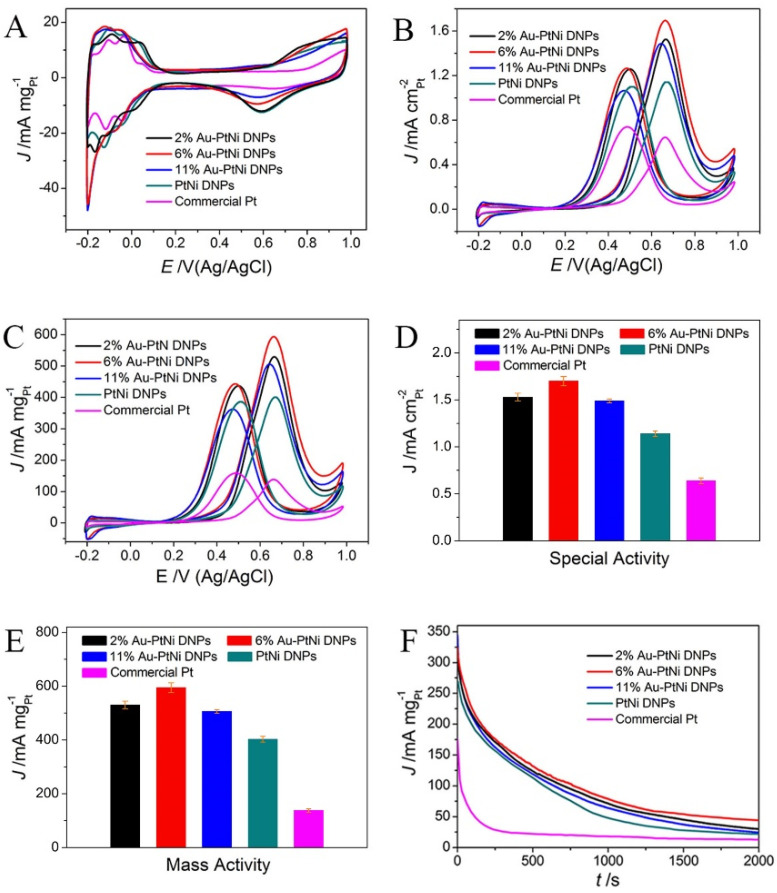
(**A**) CV curves of the 2% Au-PtNi DNPs, 6% Au-PtNi DNPs, 11% Au-PtNi DNPs, PtNi DNPs, and commercial Pt obtained in a N_2_-purged 0.5 M H_2_SO_4_ electrolyte with a scan rate of 50 mV/s. CV curves of five samples for MOR were recorded in 0.5 M H_2_SO_4_ and CH_3_OH aqueous solution at a sweep rate of 50 mV/s; the current densities were normalized by the (**B**) ECSA and (**C**) Pt mass, respectively. (**D**) Specific activities and (**E**) mass activities of five samples on peak potential. (**F**) CA curves of five samples were recorded in N_2_-purged 0.5 M H_2_SO_4_ and CH_3_OH solution at 0.6 V (vs. Ag/AgCl). For every catalyst, the electrochemical results are the mean value of more than three different tests.

The unique dendrite structure and porous shell of the prepared ternary metallic DNPs are expected to promote the MOR catalytic performance. The 2% Au-PtNi DNPs, 6% Au-PtNi DNPs, and 11% Au-PtNi DNPs were used as electrocatalysts for testing their electrocatalytic performance toward the MOR, in comparison with the PtNi DNPs and commercial Pt catalysts. Figure 4A exhibits the CV curves of the as-prepared Au-PtNi DNPs, PtNi DNPs, and commercial Pt catalysts with the mass of Pt loading (determined by ICP)-normalized current densities. The feature of the CV profiles of Au-PtNi and PtNi DNPs is similar to that of Pt with less-distinct adsorption/desorption characteristics, which is because of the presence of the dopants Au and Ni on the surface of the Pt. In the CV curves, hydrogen desorption/adsorption takes place in the range of −0.19 < E < 0.15 V (vs. Ag/AgCl), then comes the smooth double-layer charging current from 0.45 < E < 0.38 V (vs. Ag/AgCl), followed by the Pt oxidation/Pt oxide reduction at 0.38–0.99 V (vs. Ag/AgCl) [35]. The ECSA values calculated by the hydrogen desorption area in the CV profiles show that the ECSAs of 2% Au-PtNi DNPs, 6% Au-PtNi DNPs, 11% Au-PtNi DNPs, PtNi DNPs, and commercial Pt are 34.9, 34.6, 33.1, 35.0, and 21.5 m^2^/g_Pt_, respectively (Table 1), which are similar to results obtained by the CO-stripping method (the ECSAs of 2% Au-PtNi DNPs, 6% Au-PtNi DNPs, 11% Au-PtNi DNPs, PtNi DNPs, and commercial Pt are 40.2, 38.8, 37.6, 41.5, and 23.4 m^2^/g_Pt_, respectively). The ECSAs of Au-PtNi DNPs are higher than that of commercial Pt indicating the more active sites in the 3D open dendritic structure. Nevertheless, the ECSAs of Au-PtNi DNPs are lower than that of the starting PtNi DNPs, which is due to a small quantity of dopant Au in the PtNi DNPs having occupied the active sites of starting PtNi DNPs.

The catalytic activity of Au-PtNi DNPs toward the MOR was assessed in N_2_-purged 0.5 M H_2_SO_4_ and CH_3_OH aqueous solution. Figure 4B,C show the obtained CV curves with the ECSA-normalized and the Pt mass (determined by ICP)-normalizing current densities, respectively. In the positive scan direction, the current density gradually increased until a clear anodic peak appeared at ~0.7 V, which was used to evaluate the electroactivity of the catalyst for the MOR. The peak current density normalized by ECSA (specific activity) and Pt mass (mass activity) are represented in Figure 4D,E, respectively. The results show that all the Au-PtNi DNPs display a higher electrocatalytic activity than PtNi DNPs and commercial Pt toward the MOR. The specific activities of 2% Au-PtNi DNPs, 6% Au-PtNi DNPs, and 11% Au-PtNi DNPs were 1.53, 1.7, and 1.49 mA/cm_Pt_^2^, respectively. Obviously, as shown in Figure 4D, the 6% Au-PtNi DNPs exhibit the highest specific activity, which was 1.5 and 2.7 times higher than that of the PtNi DNPs (1.14 mA/cm_Pt_^2^) and commercial Pt (0.64 mA/cm_Pt_^2^), respectively. The mass activity (Figure 4E) of 2% Au-PtNi DNPs, 6% Au-PtNi DNPs, and 11% Au-PtNi DNPs are 530.4, 594.8, and 506.2 mA/mg_Pt_, indicating the 6% Au-PtNi DNPs have the highest mass activity. The mass activity of 6% Au-PtNi DNPs was 1.5 and 4.3 times higher than that of PtNi DNPs (401.8 mA/mg_Pt_) and commercial Pt (138 mA/mg_Pt_), respectively, suggesting that the 6% Au-PtNi DNPs have significant electrocatalytic activity for the MOR. A large number of recent literatures indicate that the forward peak current density (*I*_f_) and the backward current density (*I*_b_) share the same fresh methanol molecules in the MOR. The more Pt surface is covered by oxygenated species, and a lower *I*_b_ is acquired, which indicates that the *I*_f_/*I*_b_ ratio is related to the degree of oxophilicity. In other words, the *I*_f_/*I*_b_ can be applied to estimate the degree of oxophilicity for catalysts [36,37,38]. The *I*_f_/*I*_b_ results for 2% Au-PtNi DNPs, 6% Au-PtNi DNPs, 11% Au-PtNi DNPs, PtNi DNPs, and commercial Pt are 1.22, 1.34, 1.40, 1.04, and 0.87, respectively (Table 1). The higher *I*_f_/*I*_b_ values of Au-PtNi DNPs indicate that the Au-PtNi DNPs show higher oxopholicity, especially with the content of the Au dopant increasing—the oxopholicity of Au-PtNi DNPs is higher.

The electrocatalytic durability of catalysts was investigated using a CA test. Figure 4F displays the CA curves of five catalysts performed at 0.6 V (vs. Ag/AgCl) for 2000 s within N_2_-purged 0.5 M H_2_SO_4_ and a CH_3_OH electrolyte. All the catalysts exhibited a drastic decrease in current density in the starting region, which is because of the appearance of the double-layer capacitance. Then, the currents slowly decay gently due to the adsorption and accumulation of carbonaceous intermediate products on the surface of the catalysts. The results show that 6% Au-PtNi DNPs have the highest current density during a period of 2000 s among all of the tested catalysts, revealing that 6% Au-PtNi DNPs have the super electrocatalytic durability for the MOR.

**Figure 5:**
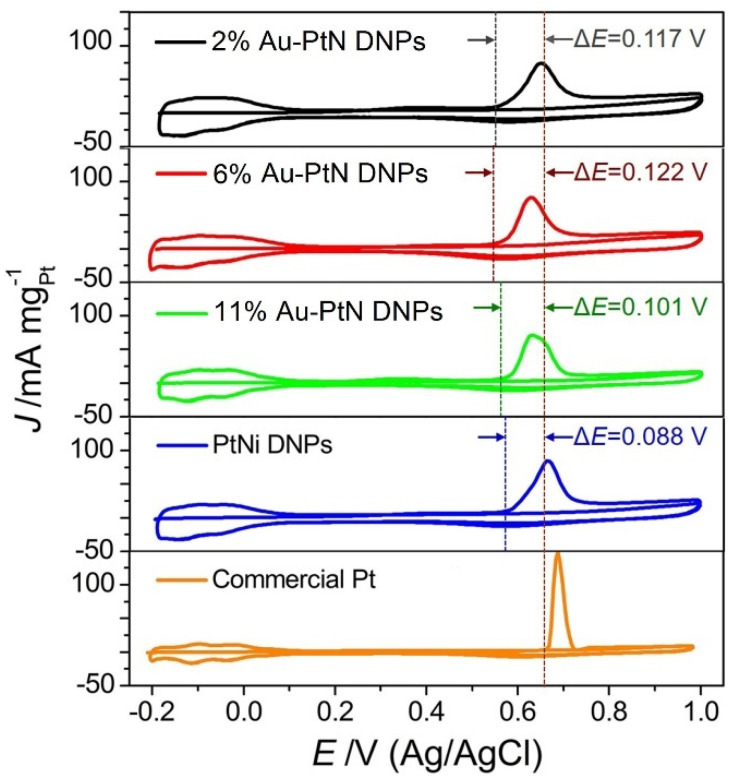
CO stripping curves of the 2% Au-PtNi DNPs, 6% Au-PtNi DNPs, 11% Au-PtNi DNPs, PtNi DNPs, and commercial Pt recorded in 0.5 M H_2_SO_4_ electrolyte with a scan rate of 50 mV/s.

CO oxidation is usually used for investigating the poisoning tolerance of catalysts under the MOR. The CO-stripping curves of five samples were performed in 0.5 M H_2_SO_4_ electrolyte at a sweep rate of 50 mV/s. As shown in Figure 5, the CO-stripping onset potential in commercial Pt is 0.658 V. For PtNi DNPs, the CO-stripping onset potential is 0.570 V, displaying a negative shift of 0.088 V as compared with that of commercial Pt. For 2% Au-PtNi DNPs, 6% Au-PtNi DNPs, and 11% Au-PtNi DNPs, the onset potentials were further moved to lower potentials of 0.541 V, 0.536 V, and 0.557 V, which show a negative shift of 0.117 V, 0.122 V, and 0.101 V, respectively, as compared with that of commercial Pt. The lowest CO-stripping onset potential of the 6% Au-PtNi DNPs, comparable with that of other catalysts, implied that CO was weakly adsorbed on the surface of the 6% Au-PtNi DNPs compared to other samples with different compositions, resulting in its excellent electrocatalytic activity toward the MOR. These analyses indicate that the prepared Au-PtNi DNPs possess super electrocatalytic durability and higher poisoning tolerance than PtNi DNPs and commercial Pt. The catalytic activity decline of PtNi DNPs is mainly due to the CO poisoning on its surface during the oxidation and/or reduction processes [35]. Generally, the electrocatalysis for the MOR on the Pt-based catalysts mainly contains two steps: Firstly, methanol adsorbs on the catalyst surface and deprotonates to generate carbonaceous intermediate species (such as Pt-COH_ads_, -CO_ads_). Secondly, water dissociates on the catalyst surface to produce hydroxyl species (-OH, Pt-OH_ads_), which interact with CO-like species to release CO_2_. The presence of Au can weaken COH_ads_/CO_ads_ binding on Pt, thus, promoting the oxidation of Pt-COH_ads_/Pt-CO_ads_ on a lower potential. In the process of electrocatalytic MOR, the less Pt-COH_ads_/Pt-CO_ads_ aggregation on the surface of Au-PtNi, as well as the easier oxidation of Pt-COH_ads_/Pt-CO_ads_, result in enhanced electroactivity activity and durability [39,40]. Among the three compositions of Au-PtNi DNPs, 6% Au-PtNi DNPs showed the best MOR performance.

Based on the results of CV, CA, and CO-stripping assessments for the electrocatalytic performance, it was indicated that the trimetallic Au-PtNi DNPs have the highest catalytic performance than the PtNi binary DNPs and commercial Pt. In our opinion, the enhanced electrocatalytic performance of the 6% Au-PtNi DNPs is based on the following factors: First, the 3D porous dendritic shell was able to greatly enlarge the surface area and benefit the reactant molecules to reach electrocatalytic active sites of AuPtNi. In addition, the synergistic “electronic effect” and “surface effect” via combining the alloying of Au, Ni, and Pt with dendritic structures is beneficial to facilitate the catalytic performance of Au-PtNi. Third, alloying the transition metal Ni with a Pt lattice could help to increase the open O sites and accelerate the adsorption of hydroxyl species to the surface of Pt [41,42], which subsequently reacts with the harmful intermediate species (CO or CO-like species) at a lower potential compared to commercial Pt [4,23]. Furthermore, the excellent durability of Au-PtNi DNPs is because of the weakened CO-like intermediate species binding on Pt when Au is doped, in which Au probably decorates the electronic structure of Pt and enhances its poisoning tolerance [23]. Taken together, the 6% Au-PtNi DNPs offer promising applications in the field of fuel cells.

## 4. Conclusions

The Au-doped PtNi DNPs with different compositions were prepared, using the PtNi DNPs as the initial material, and then doping Au via an in situ reduction of Au^3+^ in an oil bath. TEM and HRTEM images clearly suggest the formation of AuPtNi/Pt core-shell-like DNPs with a 3D porous structure. Among all the prepared Au-PtNi/Pt DNPs, the 6% Au-PtNi DNPs displayed 1.5 and 4.3 times higher mass activity than PtNi DNPs and commercial Pt, respectively. This enhanced electrocatalytic performance of the 6% Au-PtNi DNPs can be owed to the “surface effect” as well as the “electronic effect” by combining the dendritic structures and the composition of Au, Pt, and Ni. In summary, the present work not only provides a reference for the preparation of shape-controllable multi-element-doped PtM catalysts but also provides ideas for the design of super catalysts in other fields.

## Figures and Tables

**Table 1:** ICP dates, ECSA, Specific Activity (*j_a_*), Mass Activity (*j_m_*), and *I*_f_/*I*_b_ of Samples.

Sample	Au:Pt:Ni (ICP Atomic Ratio)	ECSA (m^2^/g_Pt_)	MOR Activities		
			*j*_a_ (mA/cm_Pt_^2^)	*j*_m_(mA/mg_Pt_)	*I*_f_/*I*_b_
2% Au-PtNi DNPs	2.1:92.9:5.0	34.9	1.53	530.4	1.22
6% Au-PtNi DNPs	5.6:89.6:4.8	34.6	1.70	594.8	1.34
11% Au-PtNi DNPs	11.2:85.1:3.7	33.1	1.49	506.2	1.40
PtNi DNPs	0:6:94	35.0	1.14	402.7	1.04
Commercial Pt	0:0:100	21.5	0.64	138.0	0.87

The catalysts were named 2% Au-PtNi, 6% Au-PtNi, and 11% Au-PtNi on the basis of the ICP dates.

## Data Availability

The data presented in this study are available in article and Supplementary Material.

## Supplementary Materials

The following supporting information can be downloaded at: https://www.mdpi.com/article/10.3390/nano13212855/s1, Figure S1: Particle size distribution plot and EDS pattern of 6% Au-PtNi DNPs; Figure S2: TEM image of PtNi nanodendrites; Figure S3: (A) TEM image of PtNi nanodendrites, (B) HRTEM image of the circled region in (A), Scale bare is 20 nm; Figure S4: Particle size distribution plot and EDS pattern of 2% Au-PtNi DNPs; Figure S5: Particle size distribution plot and EDS pattern of 11% Au-PtNi DNPs.
