# Supplementary material for: Au Doping PtNi Nanodendrites for Enhanced Electrocatalytic Methanol Oxidation Reaction
Source: Nanomaterials (Basel). 2023 Oct 28;13(21):2855. doi: 10.3390/nano13212855 (PMC10650142; doi:10.3390/nano13212855)
Supplement: Supplementary file 1 [file nanomaterials-13-02855-s001.zip › nanomaterials-2607262-supplementary.pdf]

Supporting Information

Au Doping PtNi Nanodendrites for Enhanced Electrocatalytic Methanol Oxidation Reaction

Shan Wang,[1] Lifeng Ma,[1] Dan Song[1], and Shengchun Yang[2, 3, 4] **

[1] Key Laboratory for Molecular Genetic Mechanisms and Intervention Research on High Altitude Disease of Tibet Autonomous Region, School of Medicine, Xizang Minzu University, No. 6 East Wenhui Road, Xianyang 712082, China

[2] Ministry of Education Key Laboratory for Non-equilibrium Synthesis and Modulation of Condensed Matter, Key Laboratory of Shaanxi for Advanced Materials and Mesoscopic Physics, State Key Laboratory for Mechanical Behavior of Materials, School of Physics, Xi'an Jiaotong University, No. 28 West Xianning Road, Xi'an 710049, China

[3] National Innovation Platform (Center) for Industry-Education Integration of Energy Storage Technology, Xi'an Jiaotong University, No. 28 West Xianning Road, Xi'an 710049, China

[4] Shaanxi Collaborative Innovation Center for Hydrogen Fuel Cell Performance Improvement, Xi'an Jiaotong University, No. 28 West Xianning Road, Xi'an 710049, China

** Corresponding author: swang@xzmu.edu.cn (S.W.);*

ysch1209@mail.xjtu.edu.cn (S.Y.)

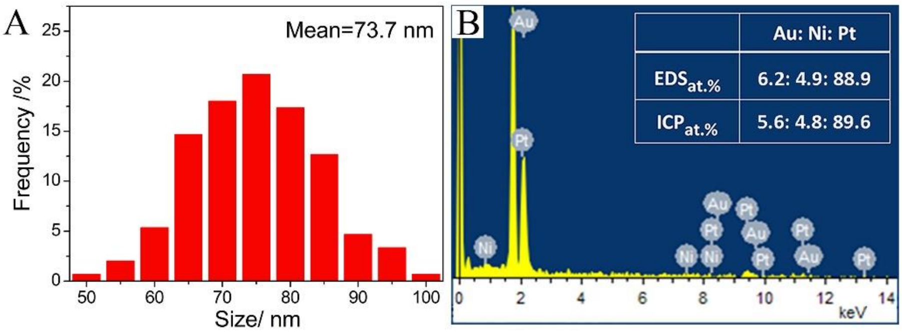

Figure S1. Particle size distribution plot and EDS pattern of 6%Au-PtNi DNPs.

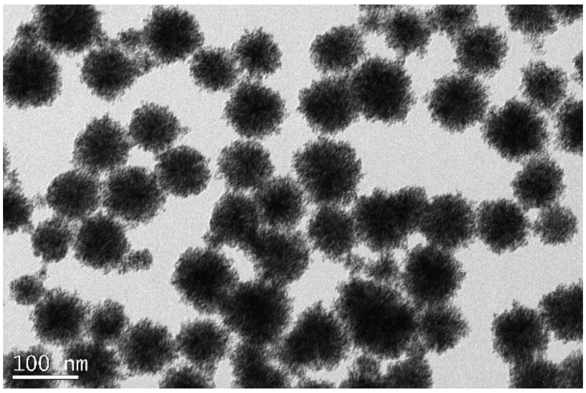

Figure S2. TEM image of PtNi nanodendrites.

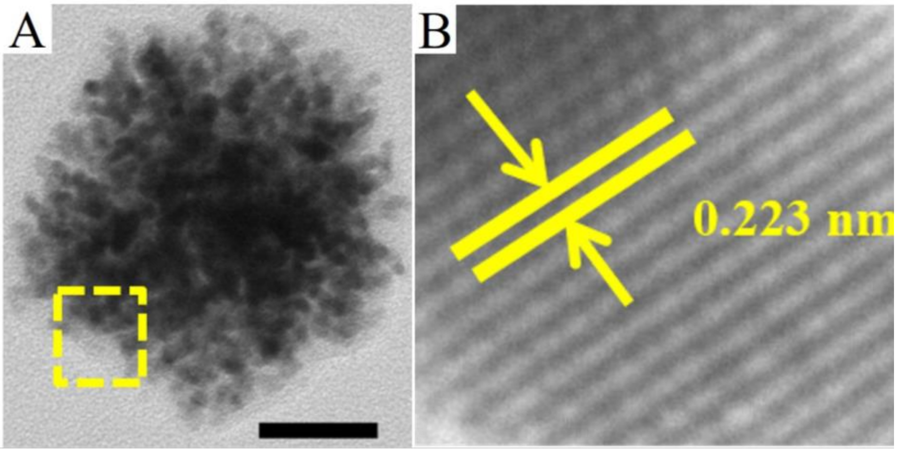

Figure S3. (A)TEM image of PtNi nanodendrites. (B) HRTEM image of the circled region in (A) [29]. Scale bare is 20 nm.

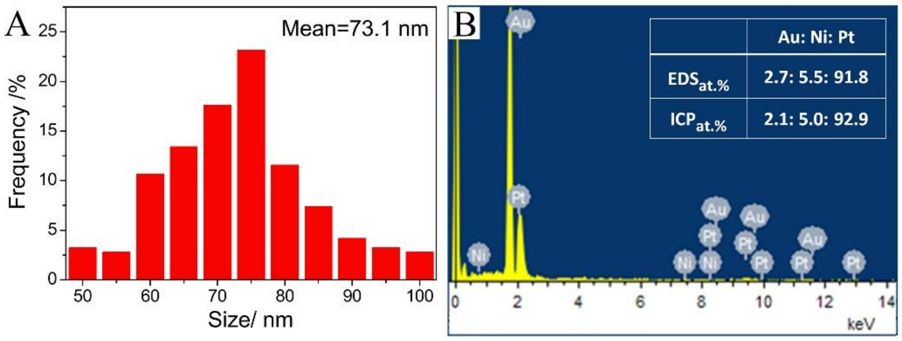

Figure S4. Particle size distribution plot and EDS pattern of 2%Au-PtNi DNPs.

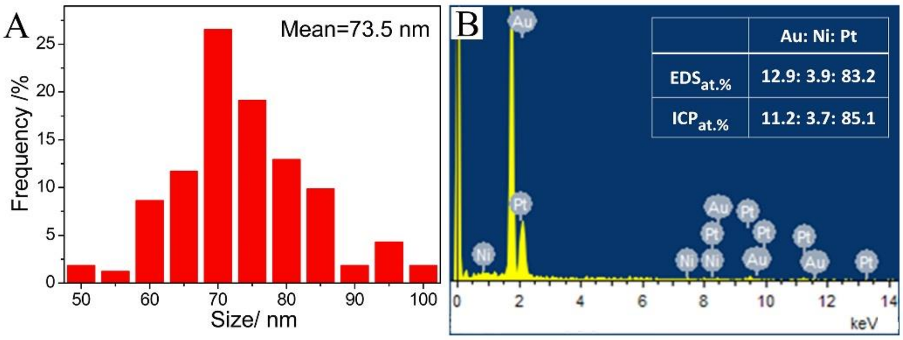

Figure S5. Particle size distribution plot and EDS pattern of 11%Au-PtNi DNPs.
